# The effect of perceived organizational fairness on the endogenous motivation of university teachers’ professional development: A study of the mediating role of trust and organizational identification

**DOI:** 10.1371/journal.pone.0317445

**Published:** 2025-01-16

**Authors:** Yangqin Wang

**Affiliations:** School of Teacher Education, Hubei University of Science and Technology, Xian’ning, China; National University of Modern Languages, PAKISTAN

## Abstract

**Objective:**

The purpose of this study is to investigate the effects of perceived organizational fairness, organizational identity, and trust on the intrinsic motivation for the professional development of university teachers. In addition, this study aims to verify the mediating role of organizational identity and trust.

**Method:**

This study adopts a quantitative research methodology to investigate the relationship between perceived organizational fairness, organizational identity, trust, and intrinsic motivation in the professional development of university teachers by constructing and validating a structural equation model.

**Result:**

The study shows that perceived organizational fairness has a significant positive effect on the intrinsic motivation for the professional development of university teachers. Moreover, it validates the mediating effects of organizational identity and trust in the relationship between perceived organizational fairness and intrinsic motivation for teachers’ professional development, shedding light on the psychological mechanisms underlying teachers’ professional development motivation.

**Conclusion:**

Organizational identity and trust not only directly influence teachers’ intrinsic motivation for professional development but also serve as mediators in the relationship between perceived organizational fairness and intrinsic motivation. These findings have important implications for university management in promoting teachers’ professional development and enhancing educational quality.

## 1. Introduction

Perceived organizational fairness refers to an individual’s evaluation of the ethical and moral dimensions of managerial behavior [1], reflecting a subjective assessment of ethical standards and inherent expectations. Initially, it was extensively studied within the domain of business management, where its impact on employee behavior and attitudes has been thoroughly explored. For instance, Masterson [2] argued that perceptions of organizational fairness are critical in fostering consumer satisfaction and loyalty. Lipponen et al. [3] identified its crucial role in organizational mergers and post-merger integration. Nurse [4] highlighted its significant impact on performance appraisal systems and employee development. Liao and Tai [5] demonstrated that trained employees’ perceptions of fairness influence their motivation, reactions, learning capacity, and the overall effectiveness of training programs. Cropanzano et al. [1] further argued that when justice is embedded as a core value within organizational management philosophies and practices, it can provide a sustained competitive advantage. Simmons [6] proposed that perceived organizational justice serves not only as a mediating variable between creativity-enhancing traits and creative performance but also plays a central role in facilitating creative output.

However, with the rapid development of contemporary higher education, the professional development of university faculty has become increasingly crucial, as it determines whether the quality of teaching and academic competitiveness can align with the high-quality, connotative development of colleges and universities. Consequently, the concept of perceived organizational fairness has gradually been integrated into the study of university faculty’s professional development. Teachers’ professional development is not only linked to the realization of individual career goals but also serves as a driving force for academic innovation and educational reform.

Despite this, the rapid growth of higher education has caused the professional development of faculty to lag significantly behind the evolving needs of education and teaching [7]. Although external incentives, such as title promotion and salary increase, can have some motivational effects, intrinsic motivation, which originates from the individual teacher and encompasses both intrinsic drive and self-directedness, is more critical for maintaining the sustained continuity and depth of their professional growth [8]. Endogenous motivation for teacher development stems from the intrinsic nature and initiative of individual teachers, emphasizing the human-centered and self-driven desire to improve, encapsulated in the sentiment “I want to develop” [9]. Currently, significant challenges in the professional development of university faculty have become increasingly apparent. First, teachers’ workloads are intensifying, and the competing demands of heavy course loads, research obligations, and administrative tasks make it difficult for them to allocate sufficient time and energy for professional growth. Second, the traditional teaching and evaluation system often emphasizes scientific research achievements, while neglecting the innovation and enhancement of teaching skills and methodologies. Additionally, in an environment characterized by limited resources and fierce competition, issues of fairness within university organizations are particularly conspicuous. Teachers may be less motivated to pursue professional development due to perceived unfairness, which encompasses not only the distribution of material resources but also career development opportunities, academic freedom, and involvement in decision-making processes.

Endogenous motivation and exogenous factors are two essential elements of teacher development, and they are interconnected and mutually influential [10]. In this context, the driving force behind teachers’ professional development should not solely rely on external incentives and requirements but should also explore ways to stimulate intrinsic motivation. Endogenous motivation refers to a psychological tendency toward a specific goal that is formed by an individual in response to both intrinsic and extrinsic motivating factors [11]. It drives individuals to act autonomously and proactively. Teachers’ endogenous motivation for professional development stems from the internalization of the social expectations and responsibilities associated with the “teacher” role, motivating them to engage in continuous learning to fulfill their professional duties and responsibilities [12]. Teachers’ learning behaviors, which are crucial for professional growth, are strongly linked to their intrinsic motivation and are influenced by their interactions with the environment [12]. For example, teachers may be motivated to learn due to a perceived need to enhance their students’ knowledge and skills, or due to a gap between expectations and the reality of students’ growth, driving them to solve practical problems in teaching [13]. Some teachers may view learning as a means to realize their life values while simultaneously promoting student growth [14]. Others may seek to enhance the meaning of their lives through self-reflection and proactive actions, demonstrating autonomy, positivity, initiative, and purposefulness. This underscores the subjectivity of teachers as educators and highlights their personal values [15]. From an individual perspective, teachers’ professional development should not be viewed solely in terms of its instrumental role but should also emphasize its reflection of the pursuit of a better life and deeper meaning. This pursuit significantly contributes to enhancing teachers’ sense of professional responsibility, moral consciousness, and life value [15]. Therefore, teachers’ professional development should extend beyond the mere instrumentality of the profession, becoming more deeply embedded in the pursuit and realization of both personal and societal values.

To overcome the current challenges in the professional development of university teachers, it is important to establish a fair and just environment at the organizational level to foster teachers’ endogenous motivation. Such an environment can help teachers feel respected and supported by the organization, enhance their sense of organizational identity and belonging, and build trust and expectations for their professional development. Cultivating a sense of organizational fairness can effectively stimulate teachers’ endogenous motivation, unlock their potential, and promote both their professional growth and the overall improvement in the quality of university education.

## 2. Theory and hypotheses

Equity theory forms the theoretical foundation for this study. It posits that an individual’s perception of fairness within an organization—specifically regarding resource distribution, decision-making processes, and outcomes—directly influences their motivation to engage in organizational activities [16]. Fairness within organizations is manifested in four principal areas.

Distributive justice, as defined by Adams [17] in 1965, focuses on whether the allocation of organizational outcomes and resources aligns with established criteria. Folger [18] further described it as an individual’s perception of the fairness of the outcomes they receive. For instance, in a university setting, the distribution of research funds, teaching resources, and promotion opportunities among faculty members would fall under distributive justice. Procedural fairness, emphasized by Hunton et al. [19], pertains to the fairness of the decision-making process and the rules that affect outcomes, granting employees a voice in the process. Leventhal [20] proposed six criteria for evaluating the fairness of procedures. In an academic context, this could involve the transparency and fairness of the process for awarding research grants, determining teaching schedules, or making tenure decisions. Interactional fairness, as described by Bies [21], relates to the respect and courtesy individuals receive in interpersonal interactions. Interactional justice refers to people’s perception of the fairness of how others behave in these interactions. In a university department, for example, the way colleagues and superiors communicate and treat each other during meetings or in day-to-day interactions would reflect interactional fairness. Informational justice, introduced by Colquitt [22], ensures that the information decision-makers rely on is honest and truthful. It primarily concerns whether decision-makers provide explanations or information regarding the procedures used and the distribution of results [23]. For instance, when a university administration makes changes to a policy, providing clear and honest communication about the reasons and implications would be an aspect of informational justice. Research has demonstrated that perceptions of organizational fairness can lead to diverse outcomes within an organization. Safari et al. [24] found that perceived organizational fairness positively influences impersonal trust within an organization. This suggests that when employees perceive fairness, they are more likely to trust the organization overall. Türkmena et al. [25] supported the importance of perceived organizational fairness in facilitating inter-organizational citizenship behaviors. In a university, this could translate to faculty members being more willing to participate in cross-departmental initiatives or contribute to the university’s reputation within the academic community. Dunaetz [26] emphasized the significance of maximizing organizational justice perceptions to ensure equitable treatment of individuals within the organization.

For faculty groups in higher education, perceptions of fairness are particularly closely tied to resource allocation and access to opportunities under distributive justice, as well as institutional rules and title evaluation systems under procedural justice [11]. Based on these insights, the following research hypotheses are proposed.

H1: Perceived organizational fairness has a significant positive effect on the endogenous motivation of university teachers’ professional development. Perceived organizational fairness encompasses an individual’s perception of the fairness of the organization’s resource allocation, decision-making processes, and outcomes, and this perception significantly impacts employees’ attitudes and behaviors. Chen et al. [27] revealed the mechanism by which the perception of organizational fairness indirectly enhances member satisfaction by increasing organizational identity and trust. Similarly, Gao and Yang’s [28] findings supported the positive correlation between a sense of organizational fairness and organizational identity, and further highlighted that organizational identity plays a partially mediating role in teachers’ work engagement. In addition, Wang’s [29] study further confirmed the positive influence of a sense of organizational fairness on individual performance and emphasized the mediating role of organizational identity in this influence process. Moreover, Guo and Liu’s [30] study found that leadership fairness had a significant positive effect on job performance, with dimensions of dedication—including organizational identity—partially mediating the relationship between perceptions of organizational fairness and job performance. Collectively, these studies provide strong evidence for the direct positive effect of perceived organizational fairness on teachers’ professional development motivation.
H2: Perceived organizational fairness positively influences organizational identity. The research conducted by the aforementioned scholars consistently demonstrates a positive relationship between perceptions of organizational fairness and organizational identity. When teachers perceive fairness within the organization, they are more likely to develop a strong identification with it, which is crucial for their integration and commitment to the organization.
H3: Organizational identity positively impacts teachers’ endogenous motivation for professional development. A strong sense of organizational identity can enhance teachers’ motivation to contribute to the organization and engage in professional development activities. It provides them with a sense of belonging and purpose within the organization, which in turn drives them to improve their professional skills and knowledge.
H4: Organizational identity mediates the effect of perceived organizational fairness on teachers’ endogenous motivation for professional development. Studies by Chi et al. [31], Deng [32], and others have shown that organizational identity can mediate the relationship between organizational fairness and teacher behaviors. It functions as an intermediary variable through which perceptions of fairness influence teachers’ motivation to develop professionally.

Existing research suggests that perceptions of organizational fairness are a key factor influencing employee behaviors and attitudes. Particularly in the field of higher education, perceived organizational fairness has a significant positive effect on the organizational citizenship behavior of university faculty [31]. Both perceived organizational fairness and trust are critical factors influencing various aspects of organizational behavior and employee attitudes. Ahmad and Huvila [33] demonstrated that favorable perceptions of organizational change can promote information sharing within an organization, with trust mediating this relationship. In the context of university teachers, this suggests that trust may play a similar mediating role in the relationship between organizational fairness and teachers’ professional development. When teachers trust the organization, they are more likely to share knowledge and collaborate, which is beneficial for their professional growth. Çetin and Güney [34] found that perceptions of organizational fairness and trust significantly influence employees’ organizational commitment behavior. For university teachers, organizational commitment is closely linked to their professional development, as it reflects their dedication to the institution and their willingness to invest in teaching and research. This research underscores the importance of both fairness and trust in shaping teachers’ long-term commitment and, consequently, their motivation to develop professionally. Erat et al. [35] explored the impact of transformational leadership and procedural justice on managerial trust and sustainable organizational identity. Although the focus was on managerial trust, their findings highlight the connection between fairness and trust in an organizational context. In the academic environment, similar relationships may exist, where fair procedures can enhance trust among teachers.

Based on the above literature, the following hypotheses are proposed:

H5: Perceived organizational fairness positively influences trust. The existing research on the relationship between organizational fairness and trust across various organizational contexts provides a foundation for this hypothesis. When teachers perceive fairness within the organization, they are more likely to develop trust in it.
H6: Trust positively impacts teachers’ endogenous motivation for professional development. This hypothesis aligns with the general understanding of the role of trust in fostering positive behaviors and motivation in the workplace. Trust can create a supportive working environment that encourages teachers to take risks, experiment with new teaching methods, and engage in professional development activities.
H7: Trust mediates the effect of perceived organizational fairness on teachers’ endogenous motivation for professional development. Existing studies on the mediating role of trust in organizational relationships suggest that it can play a similar role in the context of organizational fairness and teacher professional development. Trust functions as a bridge through which perceptions of fairness influence teachers’ motivation to develop professionally.

**Fig 1:**
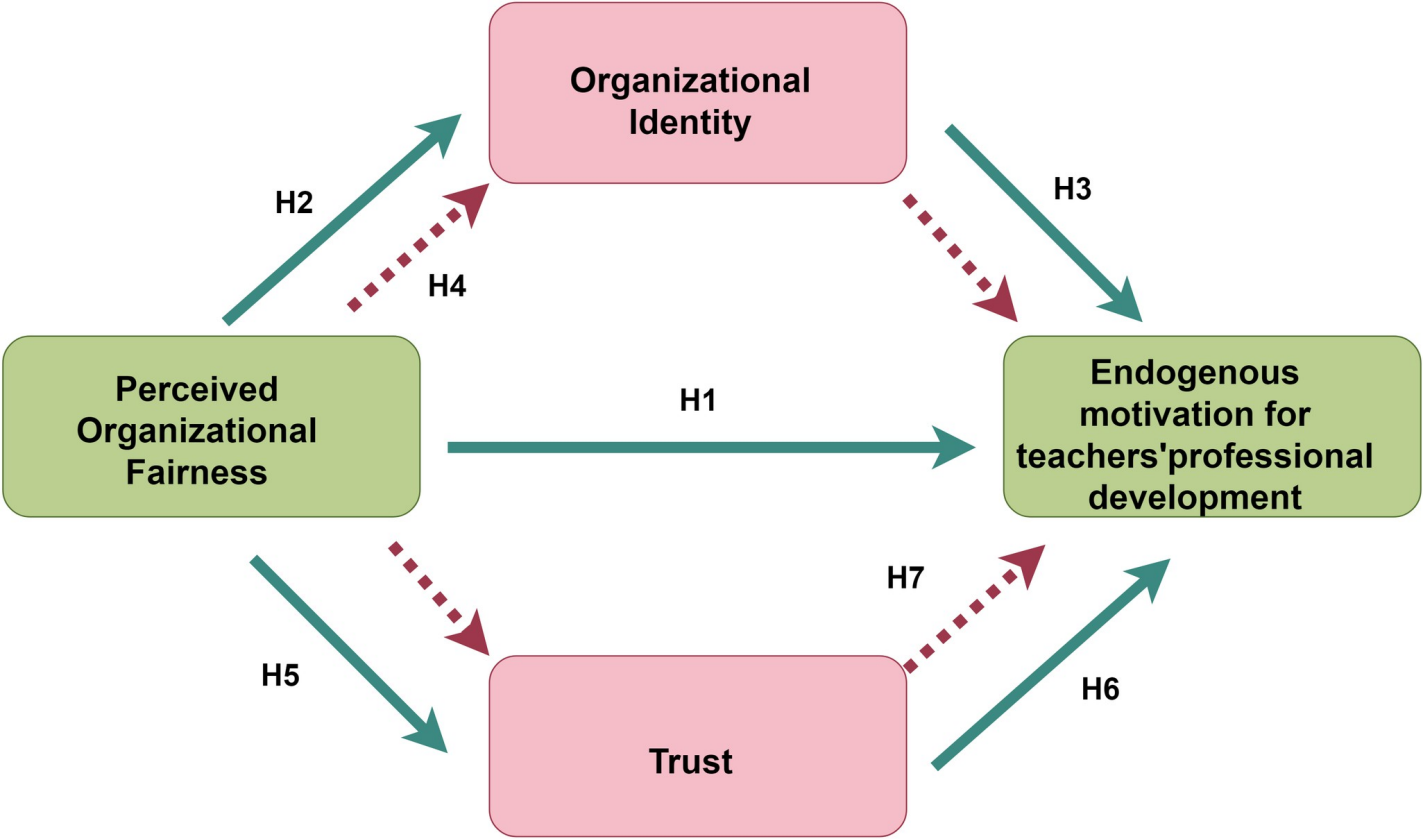
Conceptual model. (Fig 1 was drawn by Figdraw. ID: OSIAS09035).

Based on the proposed research hypotheses, a conceptual model is constructed to illustrate the relationships among perceived organizational fairness, trust, organizational identification, and teachers’ endogenous motivation for professional development. This model visually represents the theoretical framework of the study and guides the subsequent research methods and data analysis (Fig 1).

## 3. Methods

### 3.1. Survey instrument

The questionnaire for this study consists of two main parts. The first part collects demographic information about the respondents, including basic information and socio-economic characteristics. The second part focuses on evaluating the key variables in the theoretical model. The measurement items used are derived from well-established scales that have been widely recognized both domestically and internationally, ensuring the scientific validity and standardization of the research instrument. All items are based on a 5-point Likert scale, with 1 being “strongly disagree” and 5 being “strongly agree.” This study was conducted in accordance with the ethical standards of the institutional research committee and the 1964 Helsinki Declaration and its later amendments or comparable ethical standards. Informed consent was obtained from all individual participants included in the study. The study was approved by the Academic Ethics and Moral Supervision Committee of Hubei University of Science and Technology, with the following approval number: 202401004. The questionnaire will be distributed from February 2024 to April 2024. Data acquisition: Llwyh@hbust.edu.cn. Research data can be found in the S1 Table.

1. Perceived organizational fairness
  Perceived organizational fairness is a multidimensional concept. Colquitt [22] subdivided organizational fairness into four dimensions: procedural fairness, distributive fairness, interpersonal fairness, and informational fairness, with a total of 20 measurement items. Specifically, the measurement of procedural fairness involves seven items, numbered from question 1 to question 7. The measurement of distributive fairness consists of four items, numbered from question 8 to question 11. Interpersonal fairness is assessed through four items, numbered from question 12 to question 15. The measurement of informational fairness consists of five items, numbered from question 16 to question 20. Validated factor analysis showed that the four-factor model had chi-square/df = 1.90, IFI = 0.92, CFI = 0.92, Root Mean Square Error of Approximation (RMSEA) = 0.055 (0.049, 0.060).
2. Organizational identification
  Organizational identification is defined as the perceived unity and emotional belonging to an organization [36]. This definition is based on social identity theory, which suggests that people tend to identify themselves with social groups, developing positive self-concepts of the group and enhancing their own self-esteem [37]. Mael and Ashforth [38] used six-item descriptors to evaluate and make judgments about feelings toward the organization, with a scale internal consistency coefficient of 0.87.
3. Trust
  Methot et al. [39] argued that multiple relationships in the workplace create strong emotional bonds that enhance mutual trust. Trust has a significant impact on performance, drawing on Mayer and Gavin’s [40] scale to construct a trust scale, which had an internal consistency coefficient of 0.80.
4. Endogenous dynamics of teachers’ professional
  Development Sang et al. [41] developed the Teacher Professional Development Endogenous Dynamics Scale with a total reliability of 0.88. This study selected two dimensions: role responsibility and professional identity.

### 3.2. Sampling and data collection procedures

This study employed a stratified random sampling method to select 10 universities in China. The sample size calculation was conducted using the g*power program, determining the minimum sample size to be 330. Questionnaires were collected through an online platform, targeting university teachers in China. After excluding duplicate responses or incomplete data, this study obtained 514 valid questionnaires.

## 4. Data analysis

### 4.1. Statistical analysis methods

This study used SPSS 26 and AMOS 28 for statistical data analysis. First, reliability and validity tests of the scales were conducted. Second, the structural equation modeling (SEM) approach was used to construct conceptual models for path analysis of H1, H2, H3, H5, and H6. Finally, the Bootstrap method was used to test the mediating effects of H4 and H7.

### 4.2. Reliability and validity test of the scale

KMO and Bartlett’s Sphericity test were performed on the variables and the KMO value was 0.914 which was greater than 0.7 and the Bartlett’s Sphericity test sig value was less than 0.05 indicating that the data was suitable for factor analysis. The total explained variance was 68.878%, which was greater than the suggested value of 60% by Kline [42]. The 38 topics were factor analyzed using Harman’s one-way test as recommended by PODSAKOF et al. [43]. Using unrotated principal component analysis, eight factors with eigenvalues greater than 1 were extracted, of which the explained variance of factor 1 was 31.355%, which was less than the 40% criterion and passed the common method bias test, with standardized factor loadings ranging from 0.698 to 0.834 for each observed variable. The overall Cronbach’s coefficient was 0.942, and the Cronbach’s coefficients for each dimension were greater than 0.7, indicating good internal consistency across measurement topics within a single dimension [44], as shown in Table 1.

**Table 1:** Cronbach’s coefficients for each dimension.

Dimension	Cronbach alpha
Perceived organizational fairness	0.939
Organizational identity	0.870
Trust	0.890
Endogenous motivation for teacher professional development	0.853

The values of combined reliability (CR) and average variance extracted (AVE) are greater than the standardized values of 0.7 and 0.5, respectively, indicating that the convergent validity of the present model is good, as shown in Table 2. Based on the criterion proposed by Fornell and Larcker [45], a dimension is considered to have discriminant validity if the square root of its AVE is greater than its correlation coefficient with other dimensions in the model. In this study, the AVE square roots of all dimensions were greater than the correlation coefficients between them and the other dimensions, fulfilling the requirement of discriminant validity. The specific comparison results are shown in Table 3.

**Table 2:** Combined reliability (CR) and average variance extracted (AVE).

Dimensionality	Measurement problem	Factor loading	CR	AVE
Procedural fairness	Have you been able to express your views and feelings during those procedures?	0.726		
	Have you had influence over the (outcome) arrived at by those procedures?	0.777		
	Have those procedures been applied consistently?	0.725	0.900	0.563
	Have those procedures been free of bias?	0.713		
	Have those procedures been based on accurate information?	0.759		
	Have you been able to appeal the (outcome) arrived at by those procedures?	0.779		
	Have those procedures upheld ethical and moral standards?	0.771		
Distributive equity	Does your (outcome) reflect the effort you have put into your work?	0.794		
	Is your (outcome) appropriate for the work you have completed?	0.724	0.845	0.578
	Does your (outcome) reflect what you have contributed to the organization?	0.746		
	Is your (outcome) justified, given your performance?	0.775		
Interpersonal justice	Has (he/she) treated you in a polite manner?	0.781		
	Has (he/she) treated you with dignity?	0.752		
	Has (he/she) treated you with respect?	0.817	0.861	0.608
	Has (he/she) refrained from improper remarks or comments?	0.768		
Information equity	Has (he/she) been candid in (his/her) communications with you?	0.71		
	Has (he/she) explained the procedures thoroughly?	0.712		
	Were (his/her) explanations regarding the procedures reasonable?	0.724	0.844	0.521
	Has (he/she) communicated details in a timely manner?	0.745		
**Dimensionality**	**Measurement problem**	**Factor loading**	**CR**	**AVE**
Information equity	Has (he/she) seemed to tailor (his/her) communications to individuals’ specific needs?	0.716		
Organizational identification	When someone praises (name of school), it feels like a personal compliment.	0.723		
	I am very interested in what others think about (name of school).	0.672		
	When someone criticizes (name of school), it feels like a personal insult.	0.725	0.862	0.510
	When I talk about my organization, I usually say ‘we’ rather than ‘they’.	0.715		
	This school’s successes are my successes.	0.716		
	If a story in the media criticized my school, I would feel embarrassed.	0.734		
Trust	If I had my way, I wouldn’t let my coworkers have any influence over issues that are important to me.	0.828		
	I would be comfortable giving my coworkers a task or problem that was critical to me, even if I could not monitor their actions.	0.872		
	I would tell my coworkers about mistakes I’ve made on the job, even if they could damage my reputation.	0.743	0.888	0.666
	If my coworkers asked why a problem happened, I would speak freely even if I were partly to blame.	0.816		
Role responsibility	I care about the overall development of my students.	0.802		
	I would never give up on a poor student.	0.805		
	I will try to understand the problem of students with bad behavior.	0.731		
	As a teacher, my role is to assist students in discovery and inquiry.	0.834	0.883	0.602
	As a teacher, I will continue to explore the curriculum and material content.	0.698		
Professional identity	I think teachers are held in high esteem by parents.	0.729		
	I feel very happy working as a teacher.	0.78	0.813	0.593
	I feel the happiness of being a teacher.	0.799		

**Table 3:** Discriminate validity.

	Procedural fairness	Distributive equity	Interpersonal justice	Information equity	Organizational identification	Trust	Role responsibility	Professional identity
Procedural fairness	**0.750**							
Distributive equity	.517**	**0.760**						
Interpersonal justice	.344**	.460**	**0.780**					
Information equity	.456**	.533**	.398**	**0.722**				
Organizational identificatio	.407**	.449**	.382**	.433**	**0.714**			
Trust	.307**	.341**	.268**	.322**	.413**	**0.816**		
Role responsibility	.216**	.307**	.285**	.318**	.323**	.231**	**0.776**	
Professional identity	.347**	.371**	.345**	.430**	.459**	.487**	.388**	**0.770**

** Correlation is significant at the 0.01 level (2-tailed).

Note: The diagonal bold is the square root of AVE, and the lower triangle is the Pearson correlation of dimensions.

### 4.3. Structural modeling

In structural equation model evaluation, a chi-square/df ratio in the range of 0 to 3 is usually considered an indicator of good model fit. Meanwhile, similarity metrics, including the goodness-of-fit index (GFI), adjusted goodness-of-fit index (AGFI), Tucker-Lewis index (TLI), and comparative fit index (CFI), with values greater than 0.900 and closer to 1.000, indicate a high fit between the data and the model. In addition, indicators of variability, such as Root Mean Square Error of Approximation (RMSEA) and Standardized Root Mean Square Residual (SRMR), which are less than 0.080, are considered indicative of a good model fit. Based on the tests performed, the model fit indicators obtained in this study are as follows: the chi-square/df ratio is 2.475, the GFI is 0.862, the AGFI is 0.844, the TLI is 0.912, the CFI is 0.918, the RMSEA is 0.054, and the SRMR is 0.0496. These results indicate that a good fit between the sample data and the model was achieved and that the model has a high goodness of fit.

### 4.4 Hypothesis testing

#### 4.4.1. Direct effect test

**Fig 2:**
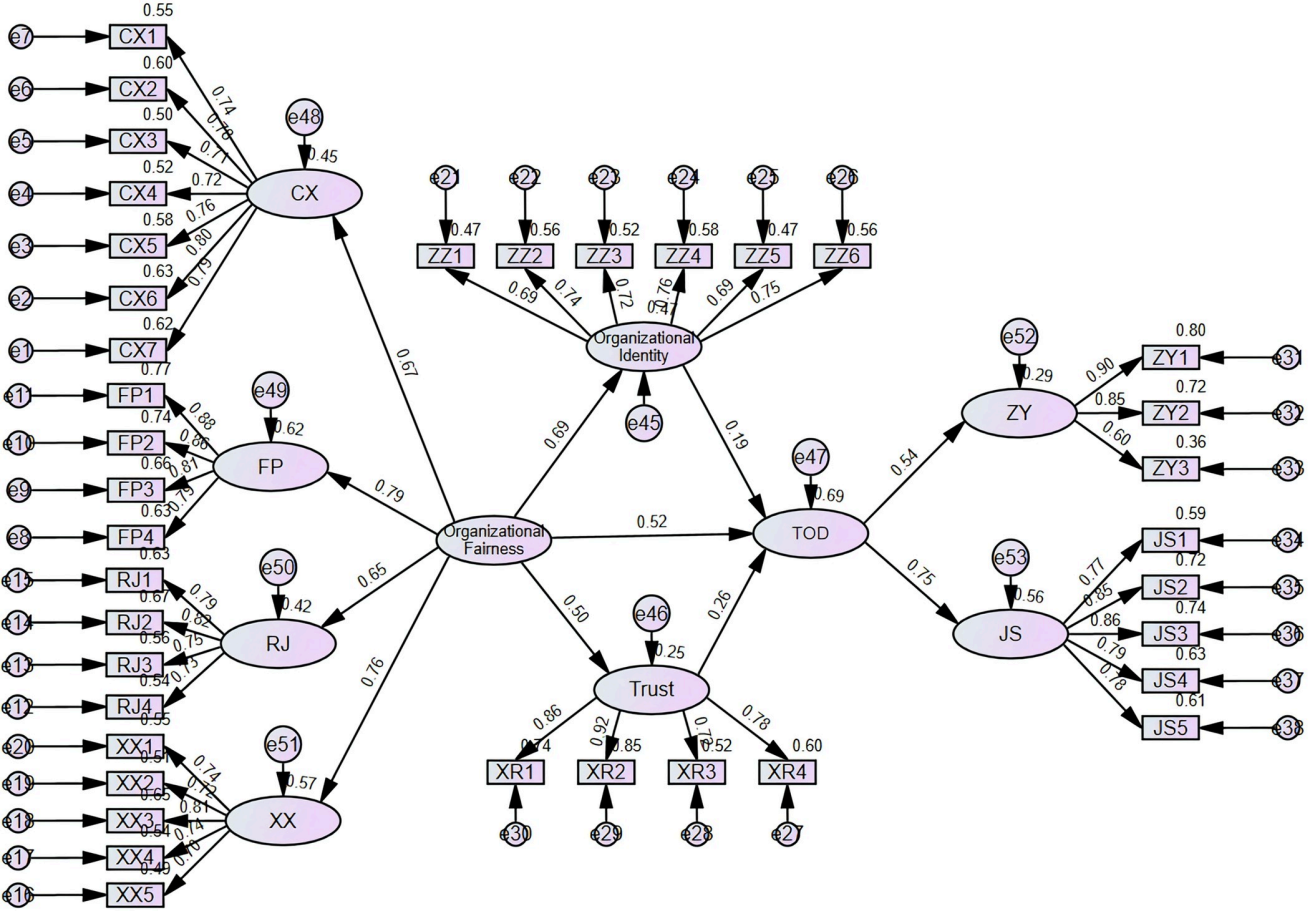
Path diagram and standardized estimate.

According to the results in Table 4 and Fig 2, the standardized path coefficients for hypotheses H1, H2, H3, H5, and H6 are 0.523, 0.687, 0.195, 0.496, and 0.264, respectively. These hypotheses are valid with a *p*-value < 0.05.

**Table 4:** Direct effect test results.

Research hypothesis	Path	Unstd.	S.E.	Z-value	Sig.	Std.	Results
H1	Perceived organizational fairness→Endogenous motivation for teachers’ professional development	0.645	0.134	4.794	<0.01	0.523	Research hypothesis holds
H2	Perceived organizational fairness→Organizational identity	1.039	0.107	9.681	<0.01	0.687	Research hypothesis holds
H3	Organizational identity→Endogenous motivation for teachers’ professional development	0.159	0.067	2.363	0.018	0.195	Research hypothesis holds
H5	Perceived organizational fairness→Trust	0.755	0.091	8.321	<0.01	0.496	Research hypothesis holds
H6	Trust→Endogenous motivation for teachers’ professional development	0.214	0.052	4.116	<0.01	0.264	Research hypothesis holds

#### 4.4.2. Mediating effect test

By resampling the sample 1000 times using the Bootstrapping method, this study tested the significance of the mediating effects of Organizational Identity (OI) and Trust. The test results are shown in Table 5 under the set condition of 95% confidence interval.

**Table 5:** Results of mediation effect analysis.

Path	Point estimates	Coefficient derived value		Bias-corrected percentile		Percentile	
		S.E.	Z	Lower	Upper	Lower	Upper
Perceived organizational fairness→Organizational identity→Endogenous motivation for teachers’ professional development	0.165	0.080	2.0625	0.041	0.307	0.038	0.303
Perceived organizational fairness→Trust→Endogenous motivation for teachers’ professional development	0.162	0.050	3.24	0.096	0.267	0.073	0.240

The mediation effect point estimate for the path “Perceived organizational fairness→Organizational identity→Endogenous motivation for teachers’ professional development” is 0.165, and the 95% confidence interval does not contain 0. This indicates that organizational identity has a significant mediating role between perceived organizational fairness and endogenous motivation for teachers’ professional development, supported by a Z-value of 2.0625, which is greater than 1.96. Similarly, the mediation effect point estimate for the path “Perceived organizational fairness→Trust→Endogenous motivation for teachers’ professional development” is 0.1622, with a 95% confidence interval that does not include 0, and a Z-value of 3.24, also greater than 1.96, indicating that trust has a significant mediating effect between perceived organizational fairness and endogenous motivation for teachers’ professional development.

## 5. Conclusion

The present study provides significant insights into the relationship between perceived organizational fairness, organizational identity, trust, and the endogenous motivation of university teachers’ professional development. The findings clearly demonstrate that organizational identity and trust have a direct, positive impact on teachers’ endogenous motivation for professional development. Teachers who exhibit a strong sense of organizational identity and trust in the organization are more likely to engage in professional development activities with greater enthusiasm and initiative [46]. This is because they experience a sense of belonging and confidence in the organization, which, in turn, drives them to pursue continuous improvement and growth in their professional careers [47].

Moreover, the mediating roles of organizational identity and trust in the relationship between perceived organizational fairness and teachers’ endogenous motivation for professional development were validated. When teachers perceive a high level of organizational fairness, it enhances their sense of organizational identity and trust, which, in turn, boosts their endogenous motivation [48]. This finding illuminates the psychological mechanism through which organizational fairness influences teachers’ motivation for professional development [49].

The results of this study have significant implications for university management. University administrators should prioritize the creation of a fair and just organizational environment, which can be achieved by ensuring transparent and equitable resource allocation, establishing fair decision-making procedures, and promoting respectful and honest interpersonal and informational interactions [50]. Such efforts will enhance teachers’ perceptions of organizational fairness, thereby strengthening their organizational identity and trust, ultimately stimulating their endogenous motivation for professional development. This, in turn, will contribute to the overall enhancement of teaching and research quality at the university [51].

In addition, this study has certain limitations. The samples were only collected from college teachers in China. Due to regional and cultural constraints, the universality of the research findings is limited. Future research could explore other potential factors that may interact with organizational fairness, identity, and trust to influence teachers’ professional development. For instance, the impact of different leadership styles on these relationships, or the role of individual characteristics such as personality traits and work values, could be further investigated [52]. Additionally, the influence of psychological empowerment and high-performance management practices on teachers’ professional development should be considered [53,54].

## Supporting information

S1 Table Questionnaire data. (XLSX)
